# Supplementary material for: Machine Learning Accelerates Crystallization for Structure Determination
Source: Angew Chem Int Ed Engl. 2026 May 4;65(25):e1218503. doi: 10.1002/anie.1218503 (PMC13266958; doi:10.1002/anie.1218503)

checkCIF/PLATON report

Structure factors have been supplied for datablock(s) lcz072

THIS REPORT IS FOR GUIDANCE ONLY. IF USED AS PART OF A REVIEW PROCEDURE FOR PUBLICATION, IT SHOULD NOT REPLACE THE EXPERTISE OF AN EXPERIENCED CRYSTALLOGRAPHIC REFEREE.

No syntax errors found. CIF dictionary Interpreting this report

Datablock: lcz072

Bond precision: C-C = 0.0123 A Wavelength=1.54184

Cell:	a=8.7939(1)	b=12.7822(3)	c=14.5413(2)
	alpha=103.013(2)	beta=104.129(1)	gamma=106.063(2)
Temperature:	100 K		

	Calculated	Reported
Volume	1446.10(5)	1446.09(5)
Space group	P -1	P -1
Hall group	-P 1	-P 1
Moiety formula	2(C15 H3 Ag3 F18 N6), C14 H7 Br O2	C15 H3 Ag3 F18 N6, 0.5(C14 H7 Br O2)
Sum formula	C44 H13 Ag6 Br F36 N12 O2	C22 H6.50 Ag3 Br0.50 F18 N6 O
Mr	2152.78	1076.40
Dx,g cm-3	2.472	2.472
Z	1	2
Mu (mm-1)	18.365	18.365
F000	1018.0	1018.0
F000'	1022.28	
h,k,lmax	10,15,18	10,15,18
Nref	5764	5407
Tmin,Tmax	0.021,0.064	0.262,1.000
Tmin'	0.003	

Correction method= # Reported T Limits: Tmin=0.262 Tmax=1.000
AbsCorr = MULTI-SCAN

Data completeness= 0.938 Theta(max)= 72.872

R(reflections)= 0.0745(5145) wR2(reflections)= 0.2104(5407)

S = 1.051 Npar= 564

The following ALERTS were generated. Each ALERT has the format
test-name_ALERT_alert-type_alert-level.
Click on the hyperlinks for more details of the test.

Alert level A

PLAT971_ALERT_2_A Check Calcd Resid. Dens. 1.01Ang From Ag01 3.90 eA-3

Author Response: This Alert is due to presence of residual density in the presence of heavy metal atom (Ag).

PLAT971_ALERT_2_A Check Calcd Resid. Dens. 1.04Ang From Ag02 3.69 eA-3

Author Response: This Alert is due to presence of residual density in the presence of heavy metal atom (Ag).

PLAT971_ALERT_2_A Check Calcd Resid. Dens. 1.04Ang From Ag03 3.54 eA-3

Author Response: This Alert is due to presence of residual density in the presence of heavy metal atom (Ag).

PLAT971_ALERT_2_A Check Calcd Resid. Dens. 1.03Ang From Ag02 3.52 eA-3

Author Response: This Alert is due to presence of residual density in the presence of heavy metal atom (Ag).

Alert level B

PLAT971_ALERT_2_B Check Calcd Resid. Dens. 1.06Ang From Ag01 3.49 eA-3

Author Response: This Alert is due to presence of residual density in the presence of heavy metal atom (Ag).

```
PLAT971_ALERT_2_B Check Calcd Resid. Dens.  1.02Ang From Ag03            3.30 eA-3
```

Author Response: This Alert is due to presence of residual density in the presence of heavy metal atom (Ag).

```
PLAT972_ALERT_2_B Check Calcd Resid. Dens.  0.88Ang From Ag01           -2.93 eA-3
```

Author Response: This Alert is due to presence of residual density in the presence of heavy metal atom (Ag) and disordered structure.

```
PLAT972_ALERT_2_B Check Calcd Resid. Dens.  0.81Ang From Ag03           -2.76 eA-3
```

Author Response: This Alert is due to presence of residual density in the presence of heavy metal atom (Ag) and disordered structure.

```
PLAT972_ALERT_2_B Check Calcd Resid. Dens.  0.88Ang From Ag02           -2.71 eA-3
```

Author Response: This Alert is due to presence of residual density in the presence of heavy metal atom (Ag) and disordered structure.

```
PLAT972_ALERT_2_B Check Calcd Resid. Dens.  0.92Ang From Ag02           -2.68 eA-3
```

Author Response: This Alert is due to presence of residual density in the presence of heavy metal atom (Ag) and disordered structure.

```
PLAT972_ALERT_2_B Check Calcd Resid. Dens.  1.01Ang From Ag01           -2.68 eA-3
```

Author Response: This Alert is due to presence of residual density in the presence of heavy metal atom (Ag) and disordered structure.

```
PLAT972_ALERT_2_B Check Calcd Resid. Dens.  0.88Ang From Ag03           -2.62 eA-3
```

Author Response: This Alert is due to presence of residual density in the presence of heavy metal atom (Ag) and disordered structure.

Alert level C

```
DIFMX02_ALERT_1_C  The maximum difference density is > 0.1*ZMAX*0.75
            The relevant atom site should be identified.
PLAT029_ALERT_3_C _diffrn_measured_fraction_theta_full value Low .      0.967 Why?
PLAT041_ALERT_1_C Calc. and Reported SumFormula    Strings  Differ     Please Check
              Calc: C44 H13 Ag6 Br F36 N12 O2
              Rep.: C22 H6.50 Ag3 Br0.50 F18 N6 O
PLAT042_ALERT_1_C Calc. and Reported MoietyFormula Strings  Differ     Please Check
              Calc: 2(C15 H3 Ag3 F18 N6), C14 H7 Br O2
```

```
              Rep.: C15 H3 Ag3 F18 N6, 0.5(C14 H7 Br O2)
PLAT088_ALERT_3_C Poor Data / Parameter Ratio ....................       9.59 Note
PLAT097_ALERT_2_C Large Reported Max.  (Positive) Residual Density       3.88 eA-3
PLAT234_ALERT_4_C Large Hirshfeld Difference F01J     --C01D     .       0.20 Ang.
PLAT342_ALERT_3_C Low Bond Precision on  C-C Bonds ..............     0.01233 Ang.
PLAT911_ALERT_3_C Missing FCF Refl Between Thmin & STh/L=    0.600        172 Report
               -5-10  1,   1 -2  3,   1 -1  3,  -9 11  3,  -3 14  3,  -8 12  4,
              -10  0  7,  -9 -3  8,  -8 -1  8, -10  0  8, -10  1  8, -10  2  8,
              -10  3  8,   6-12  9,  -8 -6  9,  -9 -3  9,  -9 -2  9,  -9 -1  9,
               -8 -1  9,  -9  0  9,  -8  0  9, -10  1  9,  -9  1  9, -10  2  9,
               -9  2  9, -10  3  9,  -8  3  9, -10  4  9,  -8 -5 10,  -8 -4 10,
                    (  142 More Missing: see the .ckf listing file)
PLAT918_ALERT_3_C Reflection(s) with I(obs) much Smaller I(calc) .          2 Check
PLAT939_ALERT_3_C Large Value of Not (SHELXL) Weight Optimized S .      10.24 Check
PLAT971_ALERT_2_C Check Calcd Resid. Dens.  1.12Ang From Ag03            1.78 eA-3
```

Author Response: This Alert is due to presence of residual density in the presence of heavy metal atom (Ag).

```
PLAT971_ALERT_2_C Check Calcd Resid. Dens.  1.11Ang From Ag02            1.78 eA-3
```

Author Response: This Alert is due to presence of residual density in the presence of heavy metal atom (Ag).

```
PLAT971_ALERT_2_C Check Calcd Resid. Dens.  1.08Ang From Ag01            1.65 eA-3
```

Author Response: This Alert is due to presence of residual density in the presence of heavy metal atom (Ag).

```
PLAT971_ALERT_2_C Check Calcd Resid. Dens.  1.06Ang From Ag03            1.63 eA-3
```

Author Response: This Alert is due to presence of residual density in the presence of heavy metal atom (Ag).

```
PLAT971_ALERT_2_C Check Calcd Resid. Dens.  1.11Ang From Ag02            1.62 eA-3
```

Author Response: This Alert is due to presence of residual density in the presence of heavy metal atom (Ag).

```
PLAT971_ALERT_2_C Check Calcd Resid. Dens.  0.91Ang From Ag01            1.52 eA-3
```

Author Response: This Alert is due to presence of residual density in the presence of heavy metal atom (Ag).

PLAT972_ALERT_2_C Check Calcd Resid. Dens. 1.74Ang From Ag01 -2.02 eA-3

Author Response: This Alert is due to presence of residual density in the presence of heavy metal atom (Ag) and disordered structure.

PLAT972_ALERT_2_C Check Calcd Resid. Dens. 0.89Ang From Ag03 -2.01 eA-3

Author Response: This Alert is due to presence of residual density in the presence of heavy metal atom (Ag) and disordered structure.

PLAT972_ALERT_2_C Check Calcd Resid. Dens. 0.84Ang From Ag02 -1.92 eA-3

Author Response: This Alert is due to presence of residual density in the presence of heavy metal atom (Ag) and disordered structure.

PLAT972_ALERT_2_C Check Calcd Resid. Dens. 0.88Ang From Ag01 -1.92 eA-3

Author Response: This Alert is due to presence of residual density in the presence of heavy metal atom (Ag) and disordered structure.

PLAT972_ALERT_2_C Check Calcd Resid. Dens. 1.13Ang From Ag01 -1.87 eA-3

Author Response: This Alert is due to presence of residual density in the presence of heavy metal atom (Ag) and disordered structure.

PLAT972_ALERT_2_C Check Calcd Resid. Dens. 1.20Ang From Ag01 -1.78 eA-3

Author Response: This Alert is due to presence of residual density in the presence of heavy metal atom (Ag) and disordered structure.

PLAT972_ALERT_2_C Check Calcd Resid. Dens. 1.11Ang From Ag02 -1.72 eA-3

Author Response: This Alert is due to presence of residual density in the presence of heavy metal atom (Ag) and disordered structure.

PLAT972_ALERT_2_C Check Calcd Resid. Dens. 0.93Ang From Ag03 -1.70 eA-3

Author Response: This Alert is due to presence of residual density in the presence of heavy metal atom (Ag) and disordered structure.

PLAT972_ALERT_2_C Check Calcd Resid. Dens. 0.73Ang From Ag02 -1.65 eA-3

Author Response: This Alert is due to presence of residual density in the presence of heavy metal atom (Ag) and disordered structure.

PLAT972_ALERT_2_C Check Calcd Resid. Dens. 0.97Ang From Ag03 -1.63 eA-3

Author Response: This Alert is due to presence of residual density in the presence of heavy metal atom (Ag) and disordered structure.

PLAT972_ALERT_2_C Check Calcd Resid. Dens. 1.03Ang From Ag01 -1.58 eA-3

Author Response: This Alert is due to presence of residual density in the presence of heavy metal atom (Ag) and disordered structure.

PLAT972_ALERT_2_C Check Calcd Resid. Dens. 0.95Ang From Ag03 -1.56 eA-3

Author Response: This Alert is due to presence of residual density in the presence of heavy metal atom (Ag) and disordered structure.

PLAT972_ALERT_2_C Check Calcd Resid. Dens. 1.32Ang From Ag01 -1.55 eA-3

Author Response: This Alert is due to presence of residual density in the presence of heavy metal atom (Ag) and disordered structure.

PLAT972_ALERT_2_C Check Calcd Resid. Dens. 1.10Ang From Ag02 -1.53 eA-3

Author Response: This Alert is due to presence of residual density in the presence of heavy metal atom (Ag) and disordered structure.

Alert level G

```
PLAT002_ALERT_2_G Number of Distance or Angle Restraints on AtSite         22 Note
PLAT003_ALERT_2_G Number of Uiso or U(i,j)  Restrained non-H-Atoms         17 Report
PLAT045_ALERT_1_G Calculated and Reported Z Differ by a Factor ...      0.500 Check
PLAT072_ALERT_2_G SHELXL First  Parameter in WGHT  Unusually Large       0.18 Report
PLAT172_ALERT_4_G The CIF-Embedded .res File Contains DFIX Records          2 Report
PLAT176_ALERT_4_G The CIF-Embedded .res File Contains SADI Records          6 Report
PLAT178_ALERT_4_G The CIF-Embedded .res File Contains SIMU Records          1 Report
PLAT186_ALERT_4_G The CIF-Embedded .res File Contains ISOR Records          1 Report
PLAT188_ALERT_3_G A Non-default SIMU Restraint Value has been used     0.0100 Report
PLAT230_ALERT_2_G Hirshfeld Test Diff for    F01I     --C01D     .        5.3 s.u.
PLAT232_ALERT_2_G Hirshfeld Test Diff (M-X)  Ag02     --N00M     .        5.7 s.u.
PLAT242_ALERT_2_G Low    'MainMol' Ueq as Compared to Neighbors of       C011 Check
PLAT242_ALERT_2_G Low    'MainMol' Ueq as Compared to Neighbors of       C017 Check
PLAT242_ALERT_2_G Low    'MainMol' Ueq as Compared to Neighbors of       C018 Check
PLAT242_ALERT_2_G Low    'MainMol' Ueq as Compared to Neighbors of       C01A Check
PLAT242_ALERT_2_G Low    'MainMol' Ueq as Compared to Neighbors of       C01C Check
PLAT242_ALERT_2_G Low    'MainMol' Ueq as Compared to Neighbors of       C01D Check
```

```
PLAT299_ALERT_4_G Atom Site Occupancy Constrained at ............        0.5 Check
              Br00    O1      O00I    C1      C2      C3      C4      C00T
              C5      C00V    C6      C015    C7      C01B    C014    C012
              C00Z    H015    H4      H014    H5      H012    H6      H7
PLAT301_ALERT_3_G Main Residue  Disorder .............(Resd    1)        14% Note
PLAT302_ALERT_4_G Anion/Solvent/Minor-Residue Disorder (Resd    2)       100% Note
PLAT434_ALERT_2_G Short Inter HL..HL Contact F00A     ..F00O     .       2.83 Ang.
                                                    -1+x,y,z  =      1_455 Check
PLAT434_ALERT_2_G Short Inter HL..HL Contact F00K     ..F3       .       2.79 Ang.
                                                  1-x,1-y,-z  =      2_665 Check
PLAT720_ALERT_4_G Number of Unusual/Non-Standard Labels .........         60 Note
              Ag01    Ag02    Ag03    Br00    F005    F006    F007    F008
              F009    F00A    F00B    F00C    F00D    N00E    N00F    F00G
              N00H    O00I    N00J    F00K    N00L    N00M    F00N    F00O
              C00P    H00P    C00Q    C00R    H00R    C00T    C00V    C015
              H015    C01B    C014    H014    C012    H012    C00U    C00W
              C00X    H00X    C00Y    C00Z    C010    C011    C013    F016
              C017    C018    F019    C01A    C01C    C01D    F01E    F01H
              F01I    F01J    F0      F01F
PLAT789_ALERT_4_G Atoms with Negative _atom_site_disorder_group  #         24 Check
PLAT811_ALERT_5_G No ADDSYM Analysis: Too Many Excluded Atoms ....          ! Info
PLAT822_ALERT_4_G CIF-embedded .res Contains Negative PART Numbers          5 Check
PLAT860_ALERT_3_G Number of Least-Squares Restraints ............         204 Note
PLAT910_ALERT_3_G Missing FCF Reflection(s) Below Theta(Min)[Deg]=       3.79 Note
                0  0  1,
PLAT912_ALERT_4_G Missing # of FCF Reflections Above STh/L=  0.600        184 Note
PLAT913_ALERT_3_G Missing # of Very Strong Reflections in FCF ....          2 Note
                1 -2  3,   1 -1  3,
PLAT941_ALERT_3_G Average HKL Measurement Multiplicity ..........         3.5 Low
PLAT969_ALERT_5_G The 'Henn et al.' R-Factor-gap value ..........      11.205 Note
              Predicted wR2: Based on SigI**2  1.88 or SHELX Weight 20.03
PLAT978_ALERT_2_G Number C-C Bonds with Positive Residual Density.          0 Info
```

---

4 **ALERT level A** = Most likely a serious problem - resolve or explain
8 **ALERT level B** = A potentially serious problem, consider carefully
31 **ALERT level C** = Check. Ensure it is not caused by an omission or oversight
33 **ALERT level G** = General information/check it is not something unexpected

4 ALERT type 1 CIF construction/syntax error, inconsistent or missing data
47 ALERT type 2 Indicator that the structure model may be wrong or deficient
12 ALERT type 3 Indicator that the structure quality may be low
11 ALERT type 4 Improvement, methodology, query or suggestion
2 ALERT type 5 Informative message, check

It is advisable to attempt to resolve as many as possible of the alerts in all categories. Often the minor alerts point to easily fixed oversights, errors and omissions in your CIF or refinement strategy, so attention to these fine details can be worthwhile. It is up to the individual to critically assess their own results and, if necessary, seek expert advice.

Validation response form

Please find below a validation response form (VRF) that can be filled in and pasted into your CIF.

```
# start Validation Reply Form
_vrf_DIFMX02_lcz072
;
PROBLEM: The maximum difference density is > 0.1*ZMAX*0.75
RESPONSE: ...
;
_vrf_PLAT029_lcz072
;
PROBLEM: _diffrn_measured_fraction_theta_full value Low .      0.967 Why?
RESPONSE: ...
;
_vrf_PLAT041_lcz072
;
PROBLEM: Calc. and Reported SumFormula    Strings  Differ     Please Check
RESPONSE: ...
;
_vrf_PLAT042_lcz072
;
PROBLEM: Calc. and Reported MoietyFormula Strings  Differ     Please Check
RESPONSE: ...
;
_vrf_PLAT088_lcz072
;
PROBLEM: Poor Data / Parameter Ratio ...................       9.59 Note
RESPONSE: ...
;
_vrf_PLAT097_lcz072
;
PROBLEM: Large Reported Max.  (Positive) Residual Density       3.88 eA-3
RESPONSE: ...
;
_vrf_PLAT234_lcz072
;
PROBLEM: Large Hirshfeld Difference F01J     --C01D     .       0.20 Ang.
RESPONSE: ...
;
_vrf_PLAT342_lcz072
;
PROBLEM: Low Bond Precision on  C-C Bonds ..............    0.01233 Ang.
RESPONSE: ...
;
_vrf_PLAT911_lcz072
;
PROBLEM: Missing FCF Refl Between Thmin & STh/L=    0.600        172 Report
RESPONSE: ...
;
_vrf_PLAT918_lcz072
;
PROBLEM: Reflection(s) with I(obs) much Smaller I(calc) .          2 Check
RESPONSE: ...
;
_vrf_PLAT939_lcz072
;
```

```
PROBLEM: Large Value of Not (SHELXL) Weight Optimized S .      10.24 Check
RESPONSE: ...
;
# end Validation Reply Form
```

PLATON version of 17/09/2025; check.def file version of 17/09/2025

---

duplicate check

No duplication found

Datablock lcz072 - ellipsoid plot

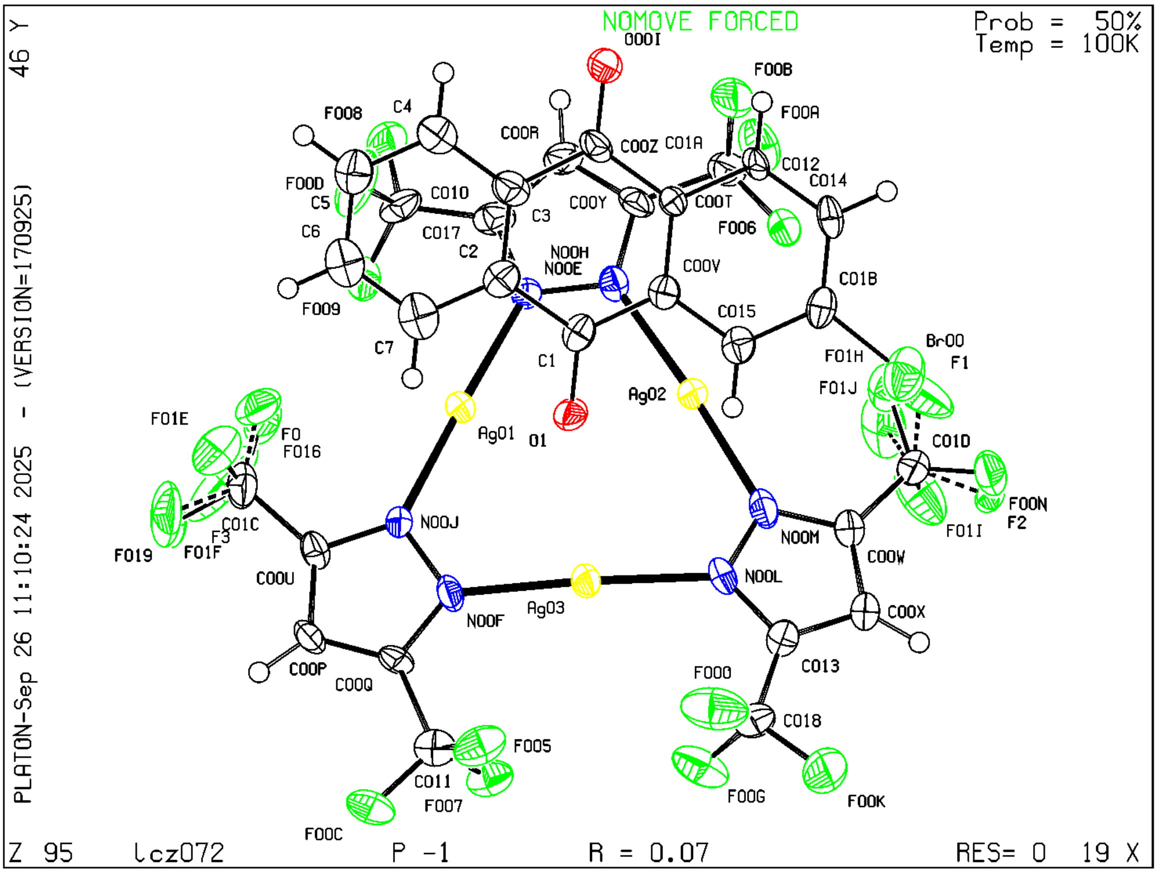

Supplement: Supplementary file 2 — Supporting File 2: anie72462‐sup‐0002‐DataFile.zip. [file ANIE-65-e1218503-s002.zip › supplementary_materials_crystallographic_data submit.zip!/cif_and_checkcif(1-114)/Ag3Pz3_72/Ag3Pz3_72_checkcif.pdf]
